# Catechins: Protective mechanism of antioxidant stress in atherosclerosis

**DOI:** 10.3389/fphar.2023.1144878

**Published:** 2023-03-24

**Authors:** Yuhan Sheng, Yizhuo Sun, Yang Tang, Yanru Yu, Jiarou Wang, Fengjie Zheng, Yuhang Li, Yan Sun

**Affiliations:** School of Traditional Chinese Medicine, Beijing University of Chinese Medicine, Beijing, China

**Keywords:** catechins, oxidative stress, atherosclerosis, lipid metabolism disorders, tea

## Abstract

Tea has long been valued for its health benefits, especially its potential to prevent and treat atherosclerosis (AS). Abnormal lipid metabolism and oxidative stress are major factors that contribute to the development of AS. Tea, which originated in China, is believed to help prevent AS. Research has shown that tea is rich in catechins, which is considered a potential source of natural antioxidants. Catechins are the most abundant antioxidants in green tea, and are considered to be the main compound responsible for tea’s antioxidant activity. The antioxidant properties of catechins are largely dependent on the structure of molecules, and the number and location of hydroxyl groups or their substituents. As an exogenous antioxidant, catechins can effectively eliminate lipid peroxidation products. They can also play an antioxidant role indirectly by activating the endogenous antioxidant system by regulating enzyme activity and signaling pathways. In this review, we summarized the preventive effect of catechin in AS, and emphasized that improving the antioxidant effect and lipid metabolism disorders of catechins is the key to managing AS.

## 1 Introduction

Atherosclerosis (AS) is characterized by lesions of the affected artery starting from the intima with accumulation of lipid and/or fibrous material. It is the underlying cause of many cardiovascular diseases, including myocardial infarction, ischaemic strokes and peripheral arterial diseases that can endanger limb viability (Libby et al., 2019).

Dyslipidemia, which is defined as derangement of the lipid profile, is one of the important factors that promote the development of atherosclerosis. It is characterized by elevated low density lipoprotein cholesterol (LDL-C) and/or decreased high density lipoprotein cholesterol (HDL-C). Examples include hypercholesterolemia and hypertriglyceridemia (Gupta et al., 2020). Oxidative stress promotes modification in lipid metabolism (Hu et al., 2019; Wójcik et al., 2019). Excessive reactive oxygen species (ROS) can destroy cellular proteins, lipids, and DNA, leading to lethal cell damage (Wójcik et al., 2021). It has been shown that elevated ROS levels promote the activation of related enzymes involved in lipid metabolism such as lipoxygenases, phospholipases, cyclooxygenases, and cytochrome p450 (Liaras et al., 2018). Most importantly, oxidative stress leads to an increase in both oxidative fragmentation and oxidative cyclization of lipid hydrocarbon chains. (Wójcik et al., 2021). In the 1950s, the presence and extent of lipids and protein oxidation products and their relationship to the severity of atherosclerotic disease were first described in humans (Forstermann et al., 2017). From the above studies, it can be concluded that abnormal lipid metabolism and oxidative stress play an important role in the formation of the AS mechanism. Therefore, improving abnormal lipid metabolism and alleviating oxidative stress is vital in the prevention and treatment of AS.

Tea is a beverage with a long history. It has always been a hot topic for scholars because it can bring significant and positive health effects (Wang et al., 2020). Tea is not only a drink, but also a traditional Chinese medicine with a long medical history. For example, tea is used as medicine in Moshizi San in Taiping Shenghui Fang (Shuhui, 2015). The health benefits of tea are largely attributed to the effects of tea polyphenol. Green tea contains high levels of tea polyphenol, most of which are catechins. They are the main components of tea polyphenols and the main reason for their antioxidant activity (Koch et al., 2020). Existing data show that catechins have antioxidant, anti-tumor and anti-inflammatory effects, suggesting that catechins have great potential in the treatment of related diseases (Bernatoniene and Kopustinskiene, 2018). Catechins can be used as chain breaking antioxidants to eliminate lipid alkoxyl and peroxyl radicals to effectively inhibit lipid peroxidation (Lambert and Elias, 2010). This provides a good therapeutic method for relieving lipid accumulation and oxidative stress in AS.

The mechanism involving catechins regulating oxidative stress to improve abnormal lipid metabolism and thus prevent AS has not been systematically mapped. In this review, we summarized the preventive effect of catechin in AS, and emphasized that improving the antioxidant effect and lipid metabolism disorders of catechins is key to managing AS. We hope to provide reference for follow-up studies of catechins in oxidative stress and abnormal lipid metabolism diseases.

## 2 Relationship between oxidative stress and lipid metabolism in AS

Alterations in lipid metabolism may lead to it becoming a risk factor and feature of AS (Poznyak et al., 2020). Low-density lipoprotein cholesterol (LDL-C) is a high risk factor for ASCVD (Stone et al., 2014). Oxidative stress is an abnormal reaction state of the antioxidant system triggered by excess free radicals in the body (Kalyanaraman, 2013). It causes lipid peroxidation, which affects the structure, fluidity, integrity of membranes, ultimately leading to destruction of cell structure and function (Juan et al., 2021).

**FIGURE 1:**
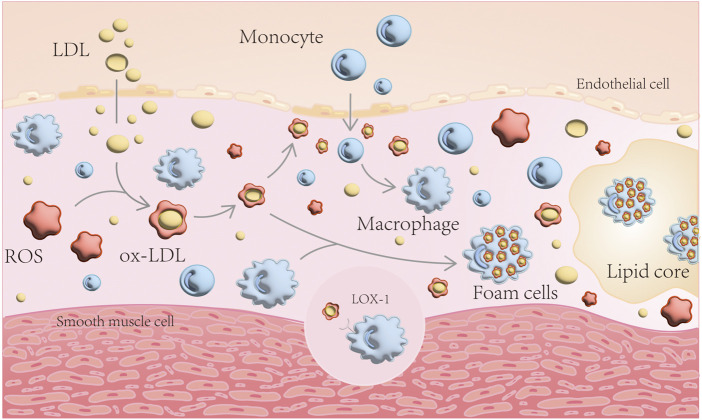
Schematic diagram of AS formation mechanism. LDL enters the subendothelium and undergoes ROS oxidation to become ox-LDL. ox-LDL damages the endothelium, allowing monocytes to enter the inner membrane and differentiate into macrophages, which engulf ox-LDL in large quantities, forming foam cells.

When vascular endothelial function is impaired, LDL enters the subendothelium and undergoes ROS oxidation to become ox-LDL. ox-LDL damages the endothelium, allowing monocytes to enter the inner membrane and differentiate into macrophages, which engulf ox-LDL in large quantities, forming foam cells, an important component of atherosclerotic plaques (Khatana et al., 2020) (Figure 1). Simultaneously, excessive accumulation of peroxidized lipids in the cell can cause endothelial dysfunction, VSMCs disorder - lipid deposition, macrophage dysfunction and foam cell formation (Hoseini et al., 2018; Cai et al., 2019; Marchio et al., 2019; Zhang et al., 2021). This chain reaction aggravates AS.

Both the lipid pathogenicity theory and the damage of endothelial cells by peroxide have confirmed the causal relationship between oxidative stress and lipid metabolism disorders in AS, and we tried to find therapeutic drugs that improve lipid metabolism disorders by regulating oxidative stress.

## 3 Basic properties and regulatory mechanisms of catechins—Protective effect against atherosclerosis

Catechins are powerful antioxidants extracted from tea. The structure of catechin is the key determinant of its free-radical scavenging and metal chelating activities. Their antioxidant activity largely depends on the number and location of hydroxyl and other chemical groups. These allow catechins to act as metal ion chelators, providing them with the ability to reduce the level of lipid peroxidation biomarkers and improve lipid metabolism disorder caused by oxidative stress. Therefore, catechins have many advantages in preventing AS. A series of experimental results show that catechins act on all aspects of the formation and progression of AS and reduce the risk of AS. Relevant studies have shown that catechins may improve AS by mobilizing endogenous antioxidant networks, including regulating enzyme activity and signaling pathways.

### 3.1 Source of catechins

Catechins are widely distributed in many foods and herbs, including apples, broad beans, pears, chocolate wine and cocoa products (Isemura, 2019). Green tea is the most abundant in catechins and is considered the leading source of all dietary sources (Ahmad and Mukhtar, 1999), ahead of chocolate, red grapes, wine and apples (Cabrera et al., 2006). According to the data of the European Food Safety Agency (EFSA), there are 126 mg of catechins in every 100 mL of green tea (Prasanth et al., 2019). The fermentation of tea is carried out by the oxidation of its own oxidase. According to the degree of fermentation, we often classify tea into four major types: Non-fermented tea, semi-fermented tea, fully fermented tea and post-fermented tea (Kondo et al., 2015). According to existing documents, tea was first consumed as a drink or medicine by the Chinese around 2737 BC, and China is now a major tea producer as well (Vuong, 2014). People in Asia have been aware of the beneficial health effects of green tea for centuries (Shixian et al., 2006). Green tea is considered as a natural plant that can maintain cardiovascular health by reducing blood cholesterol and glucose levels, and inhibiting antioxidant effects (Hara, 1994; Basu and Lucas, 2007; Shapiro et al., 2009; Roychoudhury et al., 2017). Residents in Europe, mainly the United Kingdom, drink predominantly black tea and are the largest tea consumers per day (about 540 mL) (Gardner et al., 2007). Both green and black tea are made from the fresh leaves of the tea plant, but they are processed in different ways and their catechin content is altered. Green tea is produced by drying and steaming fresh leaves, which inactivates the enzyme polyphenol oxidase, thereby protecting most of the catechins in the tea (Bartoszek et al., 2018). In contrast, in the fermentation process of black tea, catechins are oxidized and condensed to produce theaflavins and thearugins, and their content is therefore reduced (Graham, 1992).

### 3.2 Chemical properties and pharmacological effects of catechins

**FIGURE 2:**
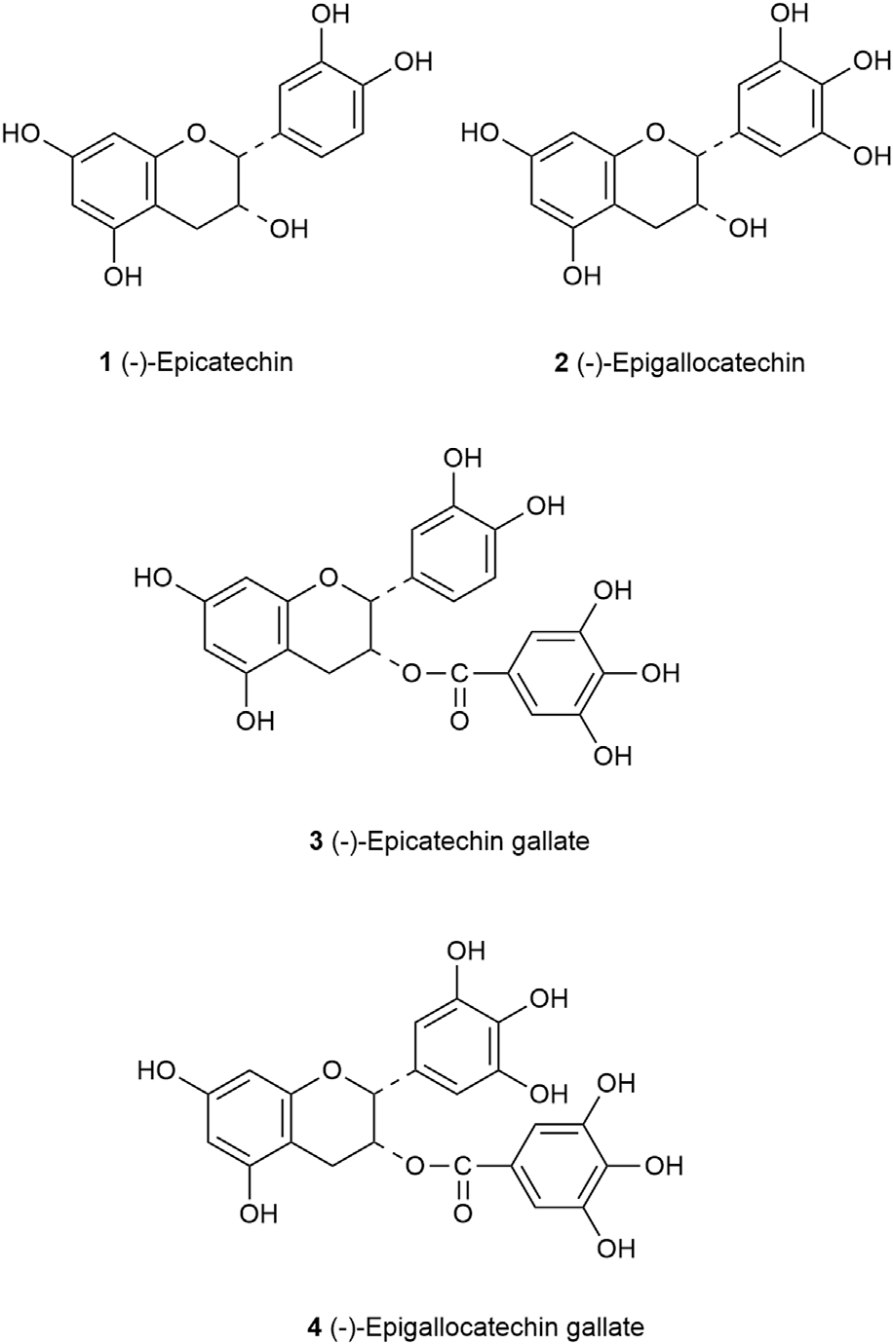
Structure of the principal catechins. EC (1) has an ortho-dihydroxyl group in the B-ring at carbons 3′and 4′and a hydroxyl group at carbon 3 on the C ring. EGC (2) has a trihydroxyl group at carbons 3′, 4′, and 5′on the B ring. ECG (3) has a gallate moiety esterified at carbon 3 of the C ring, while EGCG (4) has both a trihydroxyl group at carbons 3′, 4′, and 5′on the B ring and a gallate moiety esterified at carbon 3 on the C ring.

Catechins are a major group of flavonoids with the molecular formula C15H14O6. Studies have shown that catechins have different stability in different pH environments, which was relatively stable at pH 4–6 and changed greatly when pH was less than 3 (Musial et al., 2020). According to the different types of carbon rings, catechins are mainly divided into four groups: (−)-epicatechin (EC), (−)-epicatechin gallate (ECG), (−)-epigallocatechin (EGC) and (−)-epigallocatechin gallate (EGCG) (Peluso and Serafini, 2017). Catechins have a meta-5,7-dihydroxy group of the A ring, EC and ECG have an ortho-dihydroxyl group at carbon 3′and carbon 4′of the B ring, but EC has a hydroxyl group at carbon 2 of the C ring, ECG has a gallate moiety esterified at carbon 3 of the C ring, EGC and EGCG have a trihydroxyl group at carbon 3′, 4′and 5′of the B ring. However, EGCG has a gallate moiety esterified at carbon 3 of the C ring (Figure 2) (Higdon and Frei, 2003).

Catechins have been proved to have strong antioxidant activity. The existing literature data shows that the antioxidant activity of catechins is largely dependent on the structural of molecules, and the number and location of hydroxyl groups or their substituents (Leung et al., 2001; Chantre and Lairon, 2002). The distribution of hydroxyl groups is equally important too (Masek, 2017). The presence of one vicinal dihydroxyl group on the B ring and a galloyl group at the 3-position is essential to maintain the efficiency of the free radical scavenging capacity (Nanjo et al., 1999) In addition, Catechin chelates with metal ions to form an inactive complex, which can prevent such redox-active metal ions from catalyzing reactions and enhance their antioxidant effect. The catechol and pyrogallol groups in the B ring and the meta-5, 7-dihydroxy group in the A rings are required for the chelation of catechin with metal ions (Musial et al., 2020). EGCG rich green tea has been proven to have metal chelation properties (Thephinlap et al., 2007; Chan et al., 2016). The pyrogallol groups provide strong metal chelation of EGCG to transition metal ions that act as preventative antioxidants (Guo et al., 1996; Zhang et al., 2000).

Catechins can also effectively improve lipid peroxidation by reducing the levels of lipid peroxidation products such as malondialdehyde (MDA), 4-Hydroxynonenal (4-HNE), and F2 Isoprostane (PGF-2α). Experiments have shown that catechins can effectively reduce their level to alleviate lipid metabolism disorders caused by oxidative stress. Free radicals oxidation modifies lipids, and the final product of lipid peroxidation is MDA (Xiong et al., 2015; Zeng et al., 2021). EC can reduce the MDA content in erythrocytes in hypertensive patients (Kumar et al., 2010). An increase in Plasma MDA level was observed in N G-nitro-L-arginine methyl ester (L-NAME)-treated animals. However, after treatment with EC, MDA concentration was markedly reduced (Gomez-Guzman et al., 2011). PGF2- *a* is a recognized biomarker of oxidative stress *in vivo* and has been proved to be related to the increase of lipid peroxidation in animals and humans (Morrow et al., 1999). The 24 h urinary iso-PGF2a excretion was found to have increased after treatment with L-NAME, but excretion of iso-PGF2a returned to similar values to the control rats in the EC-treated L-NAME rats (Gomez-Guzman et al., 2011).8-Isopropane is a group of stable PGF2 *a* Isomers (Wang et al., 2007), GTE and its catechin constituents significantly reduce production of 8-iso-PGF2α after oxidative stress (Yang et al., 2016).4-HNE, an α, *ß*-unsaturated hydroxyalkenal, is a biomarker of oxidation stress (Keller et al., 2015) .4-HNE-protein adducts prompts macrophagic cells to engulf large amounts of LDL then leading to the formation of foam cells (Boléa et al., 2019). The catechins (EC, EGC, EGCG) found in white tea extracts can chelate peroxyl radicals that lead to the formation of 4-HNE (Espinosa et al., 2012). An experiment involving rats with oxidative stress induced through intraperitoneal injections of N-nitrosodimethylamine found that treatment with 0.2 mg EGCG/100 g body weight daily markedly reduced the expression of 4-HNE protein and/or mRNA levels (George et al., 2022).

### 3.3 Catechins prevent AS

Research have shown that catechins are powerful natural antioxidants (Kondo et al., 1999) that can mitigate antioxidative reactions, reducing lipid metabolism abnormalities leading to AS. According to epidemiological studies, tea consumption reduces the risk of AS and is associated with all-cause mortality (Kishimoto et al., 2020; Wang et al., 2020). Several scholars have pointed out that tea polyphenols will alleviate AS in mice by altering endothelial function, plaque size, lipid metabolism, etc., (Minatti et al., 2012; Ding et al., 2017). The main active component of tea polyphenols is catechins, which have been shown to relax blood vessels, positively regulate dyslipidemia and oxidative damage (Bernatoniene and Kopustinskiene, 2018; Wu et al., 2020).

Ox-LDL can also cause AS by inducing the regulation of oxidative stress, lipid infiltration, inflammatory response, and vascular tone by influencing nitric oxide (NO)—A versatile signaling molecule involved in maintaining metabolism and cardiovascular homeostasis in the body (Chen et al., 2018). Asymmetric dimethylarginine (ADMA), a natural occurring compound found in plasma, can inhibit nitric oxide synthase activity and has a strong inverse relationship with HDL (Lorin et al., 2013). ADMA is also inversely proportional to LDL fatty acid oxidation, which means that ADMA can regulate lipid metabolism and influence the bioavailability of NO (Paiva et al., 2006). On this basis, the dose relationship between EGCG and ADMA bivalent effect is still worth exploring. CD36 is an important intermediate in the transformation of macrophages into foam cells, and low expression of CD36 effectively delayed the development of AS (Kawai et al., 2008). A series of experiments showed that catechins effectively reduced blood lipid levels, inhibit the formation of foam cell, and resist oxidative stress (Table 1).

**TABLE 1:** Experimental studies of catechins intervention on atherosclerosis.

Reference	Treatment	Subjects	Dose	Periods	Results	Potential molecular mechnisms
Inami et al. (2007)	Polyphenon 70 S	40 healthy adult volunteers (10 men, 30 women)	each subject orally ingested Polyphenon 70 S capsules containing 500 mg of catechin	4 weeks	The plasma Ox-LDL concentration decreased significantly in the catechin group; the Ox-LDL (mg/dL)/LDL-C (U/mL) % ratio significantly decreased in the catechin group	The beneficial effect of green tea on coronary artery disease is thought to result partially from a decrease in circulating Ox-LDL.
Dower et al. (2015)	EC	37 healthy (pre)hypertensive men and women (40–80 years)	100 mg/d	4 weeks	The treatment effect of epicatechin supplementation could beneficially affect endothelial function and the development of atherosclerosis	The treatment effect of epicatechin supplementation was a significant decrease of plasma sE-selectin
Yu et al. (2021)	ECG	mices	different doses of ECGs (5, 25, and 50 mg kg−1 in w/v saline solution)	4 weeks	Serum TC, TG, LDL-C, and MDA levels were reduced; SOD activity increased	ECG reduced the progression of atherosclerosis by blocking the expression of NF-κB, and related proteins that activate the Nrf2 signaling pathway
Miltonprabu and Thangapandiyan (2015)	EGCG	rats	40 mg/kg.b.w/day	4 weeks	Pre-administration with EGCG significantly decreased the levels of plasma cholesterol, TG, FFA and PL, plasma LDL-C and VLDL-C with a significant increase in the level of HDL-C	The ability of EGCG to prevent peroxidation of membrane phospholipids. Green tea catechins are effective freer adicals scavengers and exhibits antilipid peroxidative action through their free radical-scavenging activity
					Pre-administration of EGCG along with F significantly decreased the levels of cardiac TBARS, LOOH, CD, and PC.	
Tang et al. (2006)	EGCG	rats	10 or 50 mg kg-l, dissolved in saline, i.p. Once a day	5 days	EGCG significantly attenuated the impairment of endothelium-dependent vasodilation in isolated rat aortic rings induced by native LDI concomitantly with an elevation of NO release and a decreasein serum levels of ADMA.	It is probable that the decreased level of ADMA by EGCG may be related to reduction of lipid peroxidation; Another possibility responsible for EGCG in reducing the level of ADMA is the involvement of some cytokines such as TNF-a
Friedrich et al. (2012)	EGCG	mices	Mice were fed a semi-synthetic HF diet with dietary TEAVIGO supplementation (EGCG 0.5%, EGCG 1.0%; n ¼ 12 per group)	4 days	Plasma triglycerides were reduced dose dependently by EGCG.	EGCG treatment led to a downregulation of lipogenic genes: acetyl-CoA-carboxylase, fatty acid synthase, and stearoyl-CoA desaturase
Xu et al. (2014)	EGCG	rats	IP injection of EGCG dissolved in saline (100 mg/kg body weight) once daily	12 days	Rats treated with EGCG showed a significant decrease in TC, TG, bad cholesterols, and cardiac risk ratio values and a significant increase in the level of HDL cholesterol	Administration of EGCG perhaps acted by regulating the activities of these antioxidant enzymes, such as MDA, CAT, SOD, and GPx in cardiac tissue
Chen et al. (2016)	EGCG	rats	30 or 100 mg/kg	7 days	EGCG was able to decrease the oxidative stress in hippocampi of Rs; The decreased levels of GSH, SOD, and CAT caused by Reserpine were partially and completely restored by EGCG respectively	EGCG could exert its effecting by modifying NO pathway activity; It has been reported that NO can be produced by inducible nitric oxide synthase (iNOS) and neuronal nitric oxide synthase (nNOS)
Kawai et al. (2008)	ECG	THP-1 cells	100 μg/mL	4 h	The gene expression of CD36 was significantly inhibited by the treatment with ECG	Macrophages may be the potential target of ECG in the human atherosclerotic aorta
Auger et al. (2010)	EGCG	porcine coronary arteries	100 μL	15 min	EGCG-induced concentration-dependent relaxations in porcine coronary artery	EGCG causes endothelium-dependent NO-mediated relaxations of coronary artery rings through the Akt-dependent activation of eNOS in endothelial cells
Chang et al. (2017)	catechins	Human umbilical vein endothelial cells line	50 μg/mL	4 h	Orally administrated catechins were shown to attenuate oxLDL- or PCOOH-induced vasoconstriction	Catechins decrease oxLDL- or PCOOH-induced endothelial cell apoptosis and vasoconstriction through H_2_O_2_ inhibition and eNOS restoration

#### 3.3.1 In vivo

In an intervention experiment using a high-fat diet-induced ApoE^−/−^ mice, serum TC, TG, LDL-C, and MDA levels were significantly reduced after taking ECG, while SOD activity increased. Pathological tests found that ECG reduced aortic atherosclerotic plaque size in mice (Yu et al., 2021). Animal studies have confirmed that EGCG can reduce plasma triglycerides dose dependently and inhibit cellular lipid uptake (Friedrich et al., 2012). In oxidative stress-mediated rat heart experiments, EGCG significantly reduced elevated serum cardiac markers and abnormal blood lipid metabolism caused by oxidative stress injury. At the same time, it inhibits lipid peroxidation and reduces the expression of TBARS, LOOH and CD (Miltonprabu and Thangapandiyan, 2015). EGCG also enables HFD-induced model rats to redistribute lipid levels and improve overall oxidative activity (Xu et al., 2014). Research on mice treated with reserpine to inudce excess NO and lipid peroxidation found that EGCG intervention counteracted these changes (Chen et al., 2016). EGCG can also improve vascular endothelial damage caused by LDL or ox-LDL by reducing ADMA levels (Tang et al., 2006).

#### 3.3.2 In vitro

A study of atherosclerotic mice found that ECG accumulation in macrophages specifically inhibits genes encoding CD36, an important intermediate in the transformation of macrophages into foam cells (Kawai et al., 2008). Studies have shown that EGCG can increase endothelial cell NO activity by stimulating endothelial NO synthase expression (Auger et al., 2010). In endothelial cells damaged by ox-LDL and phosphatidylcholine (PCOOH), the main metabolite of ox-LDL, catechins can also improve endothelial cell dysfunction through the above pathways and inhibit oxidative stress (Chang et al., 2017).

#### 3.3.3 Clinical trails

Studies have shown that EGCG can increase endothelial cell NO activity by stimulating endothelial NO synthase expression (Auger et al., 2010). A randomized, double blind, placebo-controlled crossover trial shows that EC may contribute to the AS protective effects through improvements in endothelial function (Dower et al., 2015).

### 3.4 Antioxidant mechanism of catechins—mobilizing endogenous antioxidant network

**FIGURE 3:**
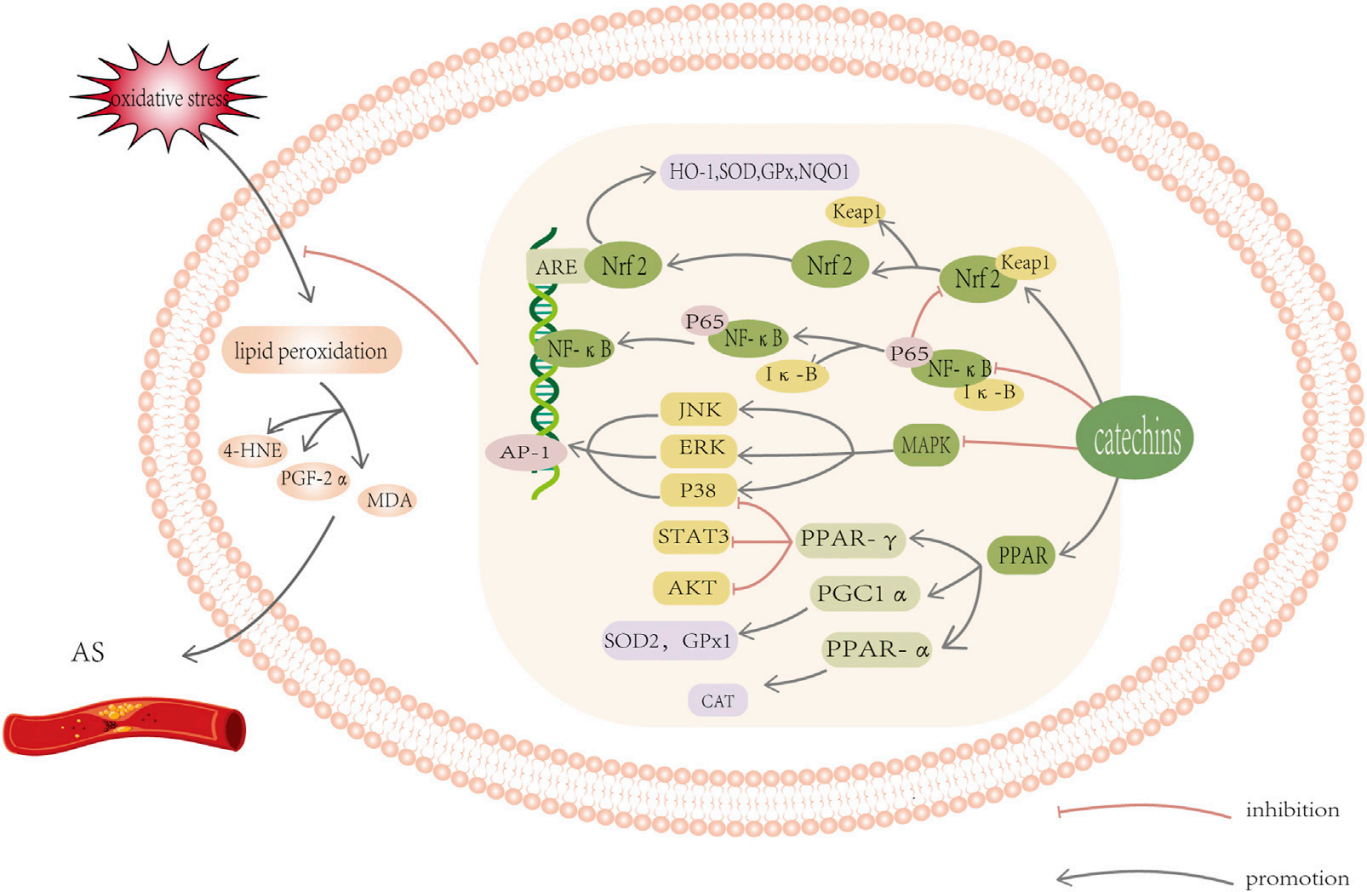
Schematic diagram of oxidative stress-related signal pathway. Catechins can induce the activation of Keap1/Nrf2/ARE signal pathway, inhibit the activation of MAPK/AP-1 pathway, and block the activation of transcription factor NF- κ B and increase PPAR γ, PGC1 *a* And PPAR *a* Protein level. These reactions all work together to help reduce oxidative stress and lipid peroxidation. For detailed explanation, please see text.

From the above statistics, we can conclude that oxidative stress can aggravate lipid metabolism disorder. Catechin, an exogenous antioxidant, is an effective scavenger of a variety of lipid peroxidation products, and can regulate oxidative stress to improve the abnormality of lipid metabolism. A series of studies had shown that catechins have good preventative and therapeutic effects on AS. Therefore, it is important to explore the antioxidant mechanism of catechins in order to mitigate lipid metabolism abnormalities. Currently, there are many discussions on the antioxidant mechanism of catechins. Here, we systematically summarized the role catechins play against oxidative stress in the endogenous antioxidant system to improve the vascular endothelial state of AS, including influencing enzyme activity and regulating signal pathways (Figure 3).

#### 3.4.1 ROS related enzymes

##### 3.4.1.1 NADPH oxidases

NADPH (Nicotinamide adenine dinucleotide phosphate) oxidases are multisubunit enzyme complexes that include p22phox and a Nox homologue and cytosolic regulatory subunits (Forstermann et al., 2017). They can produce superoxide anions *via* superoxide radical formation and play an important role in the formation of endogenous H_2_O_2_ (Bedard and Krause, 2007; Drummond et al., 2011; Sies et al., 2017). NOX and p22phox form heterodimer, which together form NOX-p22 complex in the resting state. NOX will transfer electrons to generate O_2_ , which is further converted into ROS. (Byrne et al., 2021). Endothelial NADPH oxidases are involved in proliferating and apoptosis through formation of capillary-like structures and angiogenesis (Cai, 2005). The high activity of NADPH oxidase is related to a series of proinflammatory and cytotoxic processes, which may lead to endothelial dysfunction (Steffen et al., 2008). Catechins can effectively inhibit the overexpression of NADPH oxidase. Research (Gomez-Guzman et al., 2011) found that (−)—Epicatechin treatment eliminates the increase of NADPH oxidase activity in L-NAME treated rats. The phase II metabolites of (−)—Epicatechin was believed to inhibit NADPH oxidase after observing that they prevented oxidative stress induced apoptosis of human fibroblast (Spencer et al., 2001).

##### 3.4.1.2 Xanthine oxidase

Xanthine oxidase catalyzes the conversion of hypoxanthine and generates a large number of oxygen free radicals. (Schmidt et al., 2019). Xanthine oxidoreductase initially synthesizes xanthine dehydrogenase (XDH) and is proteolytically hydrolyzed to xanthine oxidase (XO). Due to different electron receptors, although XDH and XO catalyze the same substrate, the product with opposite biochemical action is obtained: XDH reduces NAD + to NADH. However, XO cannot reduce NAD+, but catalyzes the reduced molecular oxygen to produce superoxide. Guzik et al. found that compared with non-coronary artery disease, despite similar levels of XDH, the XO protein in the blood vessels of patients with coronary artery disease is significantly increased. This indicates that the increase of XO activity contributes to the production of vascular O_2_ in coronary artery disease to a certain extent (Guzik et al., 2006). Studies have shown that catechins have inhibitory effects on XO. Lin et al. found that EGCG and tea xanthin inhibit XO to produce uric acid. Theaflavin-3,3′-digallate is the most effective XO inhibitor among a variety of tea polyphenol as a competitive inhibitor (Lin et al., 2000). Zhu et al. proved that treatment with high-dose EGCG significantly decreased the liver XO activity (Zhu et al., 2018).

##### 3.4.1.3 Cyclooxygenase 2

Studies have shown that increases in vascular superoxide content and in plasma peroxides have been observed following cardiovascular application of COX2 selective inhibitors, so COX2 is considered to suppress the level of oxidative stress. (Li et al., 2008). The increased endothelium-dependent vasoconstriction induced by acetylcholine has previously been attributed to endothelial release of prostaglandins, such as PGH2 or thromboxane A2, which are COX-derived vasoconstrictors (Auch-Schwelk et al., 1990; Duarte et al., 2002). An increase in endothelium-dependent vasoconstriction induced by acetylcholine was observed in rats aorta treated with N- nitro -L- arginine methyl ester. However, rats treated with L-NAME plus (−)—Epicatechin showed the decreased vasoconstriction response to acetylcholine and COX-2, implying that (−) epicatechin may have altered the vascular endothelial state by down-regulating COX-2 to inhibit the release of COX-derived metabolites. (Gomez-Guzman et al., 2011).

##### 3.4.1.4 Nitric oxide synthase

The indicator of endothelial dysfunction is the impairment of endothelium-dependent vasodilation mediated by NO (Augusti et al., 2008), which represents a key vasoprotective factor of the endothelium (Forstermann et al., 2017). L-arginine produce biologically active NO under that catalysis of nitric oxide synthase (NOS). Under pathological conditions, however, phagocytes are stimulated to produce excessive NO and O_2_, which react rapidly *in vivo* to form OONO- and other NO-derived oxidants (Surh et al., 2001; Higdon and Frei, 2003). Under physiological conditions, activation of endothelial nitric oxide synthase (eNOS) (a subtype of NOS) typically generates NO(Forstermann et al., 2017). In the oxidative environment, eNOS no longer produces vasoprotective NO, but instead uncouples to produce vasoinjurious O_2_ (Daiber et al., 2019). From the mechanism, deficiency of eNOS cofactor tetrahydrobiopterin (BH4) may be likely to be one of the main causes for the uncoupling of eNOS (Förstermann and Münzel, 2006; Li and Förstermann, 2013; Forstermann et al., 2017). NOX has a complex interrelationships with other ROS-producing oxidase systems. And there is more evidence that Nox-derived ROS affects the expression and activity of BH4, leading to the uncoupling of NOS(Griendling et al., 2021). Studies have found that catechins can improve phosphorylation of eNOS. When the vascular endothelium is damaged, platelets will undergo a series of activation reactions, which will lead to the production and release of pro-oxidation mediators to change the endothelial function. P-eNOS and NO bioavailability have been shown to be reduced in the activated platelet supernatant from patients with peripheral artery disease (PAD). In an experiment where human Umbilical Vein Endothelial Cells were incubated from patients with PAD and pretreated with standard epicatechin plus catechin, it was found that the bioavailability of p-eNOS and NO increased significantly. This resulted in a decrease in endothelial activation induced by activated platelets (Carnevale et al., 2014). Catechins may also improve the bioavailability of NO by reducing eNOS uncoupling. Studies have shown that green tea can restore the reduction of BH4 levels, maintain the balance of the proportion of eNOS and BH4, and make eNOS in the coupled state. Therefore, green tea reduced ROS production, reduced oxidative stress, and improved endothelial function (Faria et al., 2012).

##### 3.4.1.5 Antioxidants *in vivo*

To protect tissues from oxidation, biological systems have evolved to create multiple antioxidant systems for the removal of ROS inside cells (Parthasarathy et al., 2000). The anti-oxidation systems inherent in the human body are divided into enzymes and non-enzymes. Wherein that antioxidant enzymes comprise superoxide dismutase (SOD), catalase (CAT), and glutathione peroxidases (GPxs), and the non-enzymatic antioxidants comprise glutathione (GSH). They inhibit oxidative stress by scavenging free radicals and inactivating ROS(Chen, 2021).

Some representative phase II detoxifying enzymes include glutathione S-transferase (GST) and NAD (P) H:quinone oxidoreductase 1 (NQO1). Nrf2 can regulate the expression of these enzymes through the antioxidant-response element (ARE) and significantly enhance their antioxidant response. This process can significantly improve their antioxidant response (Kong et al., 2001).

GSH is an endogenous antioxidant that exists in two forms in the human body, reduced thiol GSH and oxidized disulfide GSSG(Raza, 2011). Depletion of GSH usually destroys the redox homeostasis of cells, leading to accumulation of ROS, which in turn triggers cell damage or even death (Li X et al., 2019). GST is involved in protecting DNA damage from oxidative stress by catalyzing the covalent binding of glutathione with hydrophobic and electrophilic substrates (Hayes et al., 2005; Chatterjee and Gupta, 2018). The Alpha class GSTs can interrupt chain of lipid peroxidation reactions by reducing hydroperoxides and detoxifying the toxic end products of lipid peroxidation. (Sharma et al., 2004). The main function of SOD is to catalyze the dismutation of superoxide anion radical into O_2_ and H_2_O_2_. They have a significant effect on the treatment of atherosclerosis by reducing the peroxidation caused by the accumulation of free radicals and maintain the metabolic balance of the body (Förstermann and Sessa, 2012; Li et al., 2014). The primary role of CAT is to catalyze the decomposition of H_2_O_2_ into H_2_O and O_2_, and protect cells from H_2_O_2_ poisoning (Wang Y et al., 2014),Overexpression of catalase reduced atherosclerosis in ApoEKO mice (Yang et al., 2004). GPx is a GSH-dependent enzyme that converts reduced GSH to oxidized GSH, and simultaneously reduces lipid hydroperoxide to the corresponding lipid alcohol or free hydrogen peroxide to water (Lubos et al., 2011). NQO1 is a homodimer flavin enzyme that catalyzes the reductions of quinones to hydroquinones through obligatory 2-electron reductions. This obligatory two-electron reduction prevents the formation of semiquinone and superoxide or H_2_O_2_ (Dinkova-Kostova and Talalay, 2010).

Reports have shown that EGCG can promote and mobilize the activities of a set of antioxidant enzymes *in vivo*, including GSH, SOD, CAT, GPX, and GST (Na and Surh, 2008). Ramesh et al., found that the activities of CAT, SOD, Gpx and GST in haemolysate and cardiac tissue samples increased significantly after being treated with EGCG (Ramesh et al., 2008). After treatment with acetaminophen (N-acetyl-p-aminophenol, APAP), EGCG increased the activities of GSH and NQO-1. In addition, the level of ROS, GSSG and TBARS in the liver decreased significantly. EGCG also increased GPxs activity, which might be responsible for the decreased ROS production during APAP metabolism (Yao et al., 2019). Polychlorinated biphenyls (PCB) can exacerbate oxidative stress in the body, and further induce inflammation of vascular endothelial cells. Studies showed that exposure of vascular endothelial cells to PCB 126 significantly increased superoxide. However, superoxide induced by PCB 126 was significantly reduced when primary vascular endothelial cells were pretreated with EGCG. Specifically, treatment of EGCG upregulated expression of antioxidant genes including GST and NQO1in a dose-dependent manner, all of which are controlled by NF-E2-related factor 2 (Nrf2) (Han et al., 2012).

#### 3.4.2 Oxidative stress related signal pathway

##### 3.4.2.1 Nrf2 pathways

The Keap1-Nrf2-ARE pathway represents one of the most important cellular defense mechanisms against oxidative stress (Bai et al., 2015; Orrù et al., 2020). Leucine zipper transcription factor (a basic region of Nrf2) can activate ARE and start a variety of antioxidant reactions to prevent oxidative stress. The Kelch-like ECH associated protein 1 (Keap1) is a receptor that affects the expression of Nrf2. Without electrophiles or oxidants, Nrf2 is located in the cytoplasm and binds to Keap1 (Kang et al., 2004). The binding of Keap1 to Nrf2 results in ubiquitin dependent proteasomal degradation under basal (reducing) conditions. Under oxidative stress, stable Nrf2 translocates to the cell nucleus and forms a heterodimer with Maf. It then interacts with ARE in target genes (Magesh et al., 2012), to drive the expression of antioxidant genes, such as NQO1, HO-1, SOD and GPx (Figuer 3) (Satoh et al., 2011; Patinen et al., 2019). Heme oxygenase 1 (HO-1) is a strong antioxidant (Araujo et al., 2012). It can increase the level of NO, reduce the level of inflammatory factors, reduce atherosclerotic plaque, and interfere with the formation and stability of plaque. In addition, HO-1 regulates cholesterol transport and plasma lipid peroxidation (Liu et al., 2012; Warner et al., 2018). Wu et al. found that after treatment with fixed concentration of 50 Amol EGCG, the level of HO-1 protein increased in a time-dependent manner. An experiment found that endothelial cell cultures cotreated with EGCG plus actinomycin D (AD) or cycloheximide (CHX) were able to completely block induction by EGCG. AD and CHX are transcriptional and translational inhibitors respectively, suggesting that EGCG most likely induced HO-1 *via de novo* RNA and protein synthesis (Wu et al., 2006). Some catechin derivatives can oxidize the cysteine thiols of Keap1, which will form disulfide bonds and release the Nfr2 (Na and Surh, 2008). For instance, under the influence of EGCG, the expression of Nrf2 decreased in cytoplasm and increased in the nucleus. Yu et al. found that ECG activated the Nrf2 and increased expression of HO-1 in ox-LDL induced VSMCs that previously had a very low expression of HO-1 and Nrf2 protein. This implies that ECG significantly ameliorated the atherosclerotic damage of VSMCs (Yu et al., 2021). Zheng et al. showed that after treatment with EGCG, nuclear accumulation of Nrf2 was significantly increased and the binding of Nrf2-ARE was also enhanced. (Zheng et al., 2012). Hence, we can conclude that EGCG can influence the mRNA expression, activity and/or protein level of Nrf2 target genes (Wang et al., 2015).

##### 3.4.2.2 PPAR pathways

Peroxisome proliferator-activated receptors (PPARs) are parts of nuclear receptor superfamily of ligand-activated transcription factors, including three member isoforms—α, β/, and γ. (Lee and Kim, 2015). PPAR- *a* is an important target for the treatment of lipid metabolism disorder, because it can regulate the expression of many lipid related genes, (Janssen et al., 2015; Kersten and Stienstra, 2017). PPAR- γ regulates target genes downstream involved in lipid production, and promotes fatty acid transport and deposition (Janani and Ranjitha Kumari, 2015; Xu et al., 2018). It is reported that pretreatment with PPAR-γ-specific antagonist saved the inhibition of activation and phosphorylation of AKT/STAT3/p38MAPK caused by PPAR-γ agonist. Therefore, PPAR -γ agonist can play an antioxidant role by means of the PPAR - γ -AKT/STAT3/p38 MAPK—Snail signaling pathway (Liu et al., 2020). After incubation with TNFα for 24 h, PPAR-γ protein levels decreased by 51% in lysates of 3T3-L1 adipocytes. EC attenuated the downregulation of PPARγ expression mediated by TNFα and reduced nuclear DNA binding (Vazquez-Prieto et al., 2012). Similarly, studies have shown that EGCG can also restore the down-regulation expression of PPAR- γ(Peng et al., 2011). Therefore, EC and EGCG may act as PPAR-γ agonists to exert antioxidant effects. In addition, PPAR-γ coactivator-1α (PGC-1α) regulates genes involved in lipid metabolism and oxidative stress (Bagattin et al., 2010; Katsouri et al., 2012; Wenz, 2013). It is also involved in the activation of PPARα Homologous. PGC1α and PPARα are key factors in antioxidant response (Fracassi et al., 2021). Research has proven that the activation of PPARα can trigger the activation of CAT, while PGC1α can regulate expression and localization of SOD2 and GPx1 (Figuer 3) (St-Pierre et al., 2006; Shin et al., 2016). The use of EC rescued the decrease in level of PGC-1α, and exhibited beneficial effects on obesity and decreased relevant cardiometabolic risk factors (Gutiérrez-Salmeán et al., 2014). Marinovic et al. demonstrated that EGC and EC can indirectly activate PPARα and reduce hepatic steatosis (Marinovic et al., 2022). Unfortunately, there are insufficient reports on the role of PPAR pathway in oxidative stress with catechins. Its role in ROS metabolism too has not been explored to a large extent.

##### 3.4.2.3 MAPK pathways

The MAPK (mitogen-activated protein kinase) signaling cascades involving MAPKs ERK (extracellular signal regulated kinase), JNK (c-Jun N-terminal kinase) and p38 MAPK may play an important role in atherosclerosis and vascular restenosis (Muslin, 2008). Inhibition of the cascade is believed to protect cells from oxidative stress. Evidence suggests that when JNK, ERK, and p38 proteins are activated, ROS level increases, leading to oxidative stress and subsequently apoptosis (Kong et al., 2021). Specifically, the JNK pathway has been demonstrated to be part of oxidative stress responses in tumors, suggesting that inhibition of JNK signaling may be helpful to prevent several ROS-induced metabolic diseases (Li C et al., 2019). Activation of AP-1, a transcription factor, occurs through the MAPK pathway. Its activity is influenced by the intracellular redox environment, including the level of ROS and antioxidants (Figuer 3) (Higdon, J. V. and Frei, B., 2003; Nomura et al., 2000). EGCG can minimize the damage to endothelial cells and reduce IL-6 and TNF-α by inhibiting AP-1 activity (Riegsecker et al., 2013; Wang Z M et al., 2014). Catechins seem to inhibit AP-1 activity through inhibiting kinases in the MAPK pathway, such as JNK and Erks (Katiyar et al., 2001). EGCG was observed to significantly prevent thrombin-induced caspase 3 activation and apoptosis by suppressing JNK phosphorylation (He et al., 2015). It also inhibited the production of plasminogen activator inhibitor-1 mediated by TNFα and reduced ERK1/2 phosphorylation (Cao et al., 2013). Treatment with a standardized green tea polyphenol decoction containing 65% EGCG reduce the phosphorylation levels of c-Jun and Erk1/2 (Lu et al., 2006).

##### 3.4.2.4 NF-κB pathways

The molecular signaling pathway regulated by catechins is responsible for its pro-apoptotic and anti-proliferative characteristics. One of which is the inhibition of a key oxidative stress-sensitive transcription factor -nuclear factor-κB (NF-κB) (Khan and Mukhtar, 2013; Musial et al., 2020). After exposure to oxidative and inflammatory stimuli, I κ B kinase (IKK) is activated, leading to IKK signalsome phosphorylation, which are subsequently degraded by the proteasome. Then NF-κB translocates to the nucleus, where it binds to specific promoter regions and initiates transcription. (Karin, 1999; Surh, 2003). In addition, NF-κB may aggravate oxidative stress by influencing the Nrf2 signaling pathway. Being a protein downstream of NF-κB, the research have shown that p65 may exert conflicting effects in the Nrf2 signaling pathway by accelerating peroxidation, leading to abnormal cell proliferation (Figuer 3) (Yang et al., 2020). Catechins, especially EGCG, can block the activation of NF-κB (Varilek et al., 2001) by many pro-inflammatory stimuli and inhibit the activity of I κ B kinase β (IKKβ, the key kinase for activating NF-kB pathway) (Youn et al., 2006). It was found that EGCG can reduce p65 expression induced by PCB (polychlorinated biphenyls) 126 and down-regulate the expression of NF-κB regulated genes, further suppressing endothelial cells inflammation (Liu et al., 2016). Another experiment discovered that ECG inhibited the phosphorylation of p65 in the NF-κB pathway, and reduce the lipid disorder and atherosclerotic lesions in ApoE^−/−^ mice induced by high fat diet (Yu et al., 2021).

## 4 Potential problems of catechin application

Many studies have proven that catechins are protective against AS and are effective natural antioxidants. However, there are still a few limitations in place such as metabolite activity and low bioavailability.

Because catechins are rapidly and extensively metabolized, *in vitro* experiments data and the biological activity of catechins metabolites are often questioned. It is hence particularly important to demonstrate catechins antioxidant activity *in vivo*. Catechins have been found to experienced considerable biotransformation *in vivo*, and their main metabolic pathways are methylation, glucuronidation, sulfation and ring-fission metabolism. (Yang et al., 2002; Feng, 2006). EGCG metabolites and metabolites produced from EC or ECG are proven to have stronger free radical scavenging power than parental catechins (Takagaki et al., 2011). The 30–and 40 -monomethyl ethers of EC can inhibit NADPH oxidase to increase NO in endothelial cells, thus reducing oxidative stress (Steffen et al., 2008). These evidence suggests that catechin metabolites can maintain the antioxidant capacity of their parent compounds. Another metabolic pathway includes the degradation of catechins. Liver and intestine are the backbone of the metabolization and absorption of catechins (Feng, 2006). Besides intestinal and liver metabolites, Sang et al. also found metabolites in colon bacteria (Sang et al., 2008). Investigation found that catechins not metabolized in the upper intestine were transported to the lower intestine through intestinal microflora (Roowi et al., 2010). Ottaviani et al. found that 70% of the ingested (¡)-epicatechin was absorbed by the lower intestinal after catabolism of intestinal microflora. (Ottaviani et al., 2016). Therefore, there is great research potential in intestinal microbiota to improve production and hence the bioavailability of catechin metabolites. It is also important to continue studying the antioxidant effect of metabolites to find the optimal condition for catechins to play an antioxidant role better in the local intestinal.

Tea polyphenols are susceptible to degradation under environmental stresses or digestive circumstances, such as alkaline pH and high temperature. In addition, the low bioavailability of catechins also due to degradation and metabolism in the gastrointestinal tract, poor membrane permeability, and pre-systemic hepatic clearance (Ye and Augustin, 2019; Sabaghi et al., 2021). The development of new agents, such as nanoparticles, may become an effective way to solve this problem in the future. Recently, studies found that nanomaterials based on carbon, nanozymes, and nanomedicine could improve stability of antioxidant treatments and further upgrade the antioxidant effect. For instance, nitrogen-doped carbon nanodots ionogels (Rizzo et al., 2018), Mn (3) O (4) nanozymes (Yao et al., 2018), and colloidal-stable nanotherapeutics made of bioadhesive chitosan materials that are suitable for oral delivery (Han et al., 2019). Green nanoparticles (GNPs) prepared by Yang et al. using TP in green tea as the monomer have strong free radical scavenging ability and oxidation resistance. The research provides a new green strategy for making safe and effective antioxidants. (Yang et al., 2021). It has been reported that synergistic effects of the combination of EGCG and fish oil. The presence of fish oil increased the bioavailability of EGCG (Giunta et al., 2010). Furthermore, using broccoli byproducts as the matrix for co-delivery of EGCG and fish oil could prevent the degradation of EGCG in the upper gastrointestinal tract can thus be metabolized by the microorganisms in the lower gut, leading to an increase in EGCG bioavailability (Shi et al., 2020). In addition, the combination of catechins with other drugs that show synergistic effects may be a promising approach, such as catechins showing good synergy with some conventional anticancer drugs (Cai et al., 2018).

Moreover, under certain conditions, catechins may have both prooxidative or toxic effects. The dual antioxidant and pro-oxidant functions of catechins depend primarily on the dose level and the biological context. In a safety study that examined genetic, acute, and short-term toxicity of EGCG, a no-adverse effect level (NOAEL) of 500 mg/kg/day of EGCG was determined (Isbrucker et al., 2006). Some European regulators have suggested that the tolerable upper intake level of EGCG should be 300 mg per day for humans (Yates et al., 2017). Tian et al. found that at 0.04%, TP promotes the oxidation of protein in emulsions with proteins at the interface, but still has a certain antioxidant effect on aqueous phase proteins. It is possible to optimize the TP level of foods or beverages based on emulsion to achieve the best antioxidant activity (Tian et al., 2022).

## 5 Conclusion

With the aging of the general population and the increase in chronic diseases such as hypertension and diabetes, the incidence rate of atherosclerosis further increase. Atherosclerosis has no obvious early symptoms. When the disease progresses to a higher stage with age, symptoms of atherosclerosis will appear. Therefore, it is very important to seek preventive diet or drugs, and the strategy of prevention before disease will greatly reduce hospital costs and other economic burdens of patients. The development of natural products to prevent AS has scientific significance and application value. At the same time, the discovery of lipid oxidation products implies that oxidative stress promotes the change of lipid metabolism, which provides a new idea for the treatment of diseases with abnormal lipid metabolism.

Tea, especially unfermented green tea, is rich in catechins, which have antioxidation and improve lipid metabolism disorders. The health benefits of tea are largely attributed to the effects of catechins. However, catechins correspond to a variety of targets and act through different signaling pathways. Due to the pleiotropic effects of catechins, more definitive studies on their biological functions and anti-atherosclerotic mechanisms are lacking before their clinical application. Current studies have not systematically revealed the mechanism of catechins in anti-oxidative stress to regulate abnormal lipid metabolism in AS. Therefore, we hope to clarify the therapeutic effect of catechin in AS by combing the mechanism of catechin regulating oxidative stress and improving abnormal lipid metabolism. This study will provide a reference for the subsequent development of catechin as AS adjuvant drugs.

Catechins play an antioxidant role in many ways, namely, by balancing enzyme activity and regulating signal pathways. They inhibit NADPH oxidase, XO, COX2, NOS, and other enzymes that produce ROS and activate antioxidants in the body, such as GSH, SOD, CAT, GPX, GST, NQO1, to significantly improve the antioxidant response. Concurrently, catechins induce the activation of Keap1/Nrf2/ARE signal pathway, inhibit the activation of MAPK/AP-1 pathway, and block the activation of transcription factor NF- κB and increase PPAR γ, PGC1 *a* And PPAR *a* Protein level. These reactions all work together to help reduce oxidative stress.

It is noteworthy to point out that there are still many limiting factors for the application of catechins, such as prooxidative and toxic effects under certain conditions, the dubious activity of its metabolites and low bioavailability. Determining the safe dose of catechin and finding the biological environment that can exert the best antioxidant activity of catechin are effective methods to overcome the pro-oxidative side effects of catechin. Promoting the catabolism of catechins by intestinal flora can enhance the absorption and utilization of the host. Isolation and identification of microorganisms and microbial metabolites with the ability to catabolize the active catechins may be one of the methods to improve the utilization of catechins. The development of new preparations of catechins based on nanomaterials greatly improves their antioxidant stability. The combination of catechin with other bioactive dietary compounds and disease treatment drugs can play a synergistic effect of promoting the absorption and utilization of both sides. All these provides a new idea for solving the problem of low bioavailability of catechins.

Current research on catechins focuses on functional and metabolic studies. In the future research, the physiological function of catechins can be combined with their chemical structure and *in vivo* process. More clinical trials can be carried out to further verify the role of catechins in the prevention and treatment of AS. Studies on the pharmacokinetics and pharmacodynamics will be the focus of the application of catechins in AS. In order to improve the clinical application of catechins, the combination of catechins with existing AS drugs may become a direction of research on AS treatment. The potential combination of pharmaceutical and nutritional levels is able to establish a more effective treatment regimen.

More researches are needed to elucidate the antioxidant mechanism of catechins. Despite its limitations, we can effectively conclude that regular intake of an appropriate amount of tea can regulate the antioxidant capacity of the human body, improve lipid metabolism, and hence prevent atherosclerosis.
